# Serologic Evidence of Recent Infection with Highly Pathogenic Avian Influenza A(H5) Virus Among Dairy Workers — Michigan and Colorado, June–August 2024

**DOI:** 10.15585/mmwr.mm7344a3

**Published:** 2024-11-07

**Authors:** Alexandra M. Mellis, Joseph Coyle, Kristen E. Marshall, Aaron M. Frutos, Jordan Singleton, Cara Drehoff, Angiezel Merced-Morales, H. Pamela Pagano, Rachel O. Alade, Elizabeth B. White, Emma K. Noble, Crystal Holiday, Feng Liu, Stacie Jefferson, Zhu-Nan Li, F. Liani Gross, Sonja J. Olsen, Vivien G. Dugan, Carrie Reed, Sascha Ellington, Sophia Montoya, Allison Kohnen, Ginger Stringer, Nisha Alden, Peter Blank, Derick Chia, Natasha Bagdasarian, Rachel Herlihy, Sarah Lyon-Callo, Min Z. Levine

**Affiliations:** ^1^Influenza Division, National Center for Immunization and Respiratory Diseases, CDC; ^2^Michigan Department of Health and Human Services; ^3^Colorado Department of Public Health and Environment; ^4^Office of Readiness and Response, CDC; ^5^Epidemic Intelligence Service, CDC; ^6^Coronavirus and Other Respiratory Viruses Division, National Center for Immunization and Respiratory Diseases, CDC; ^7^Division of Birth Defects and Infant Disorders, National Center for Birth Defects and Developmental Disabilities, CDC.

**Summary**

**What is already known about this topic?**

Infections with highly pathogenic avian influenza (HPAI) A(H5) viruses have been detected sporadically in dairy farm workers in the United States since April 2024. Public health response efforts include active monitoring of workers exposed to HPAI A(H5) virus for illness.

**What is added by this report?**

Health officials conducted surveys and serologic testing to identify recent HPAI A(H5) infections among dairy workers in two states. Serologic testing indicated that 7% of participating dairy workers had evidence of recent infection with HPAI A(H5) virus.

**What are the implications for public health practice?**

The findings support the need for active monitoring of exposed workers and testing to detect and treat HPAI A(H5) infections, including those in persons with very mild symptoms. These efforts should be coupled with farmworker education about infection risks and prevention measures.

## Abstract

Since April 2024, sporadic infections with highly pathogenic avian influenza (HPAI) A(H5) viruses have been detected among dairy farm workers in the United States. To date, infections have mostly been detected through worker monitoring, and have been mild despite the possibility of more severe illness. During June–August 2024, CDC collaborated with the Michigan Department of Health and Human Services and the Colorado Department of Public Health and Environment to implement cross-sectional serologic surveys to ascertain the prevalence of recent infection with HPAI A(H5) virus among dairy workers. In both states, a convenience sample of persons who work in dairies was interviewed, and blood specimens were collected. Among 115 persons, eight (7%; 95% CI = 3.6%–13.1%) had serologic evidence of recent infection with A(H5) virus; all reported milking cows or cleaning the milking parlor. Among persons with serologic evidence of infection, four recalled being ill around the time cows were ill; symptoms began before or within a few days of A(H5) virus detections among cows. This finding supports the need to identify and implement strategies to prevent transmission among dairy cattle to reduce worker exposures and for education and outreach to dairy workers concerning prevention, symptoms, and where to seek medical care if the workers develop symptoms. Timely identification of infected herds can support rapid initiation of monitoring, testing, and treatment for human illness, including mild illness, among exposed dairy workers.

## Introduction

Highly pathogenic avian influenza (HPAI) A(H5) viruses have been circulating among animals worldwide since 1997, with sporadic human infections, primarily associated with exposure to infected poultry.* In March 2024, HPAI A(H5) clade 2.3.4.4.b B3.13 virus was first detected in dairy cattle in the United States, a novel animal reservoir; the first human infection in a dairy worker was detected in Texas in April 2024.^†^ In response to the initial human infection, enhanced surveillance of dairy herds and poultry facilities in the United States has led to the detection of additional, sporadic human infections among workers in these industries.^§^ Despite ongoing efforts to monitor dairy workers for illness, test for HPAI A(H5), and offer antiviral treatment, several factors, including absence of serious illness to date, barriers to testing and reporting, and reluctance of some farms and workers to participate in monitoring efforts, have prevented gaining a full understanding of the extent of cow-to-human transmission.

CDC supported the Michigan Department of Health and Human Services (MDHHS) and the Colorado Department of Public Health and Environment (CDPHE) in conducting seroprevalence investigations among workers on dairies known to be infected with HPAI A(H5) viruses. The goals were to measure HPAI A(H5) seroprevalence, to identify risk factors for infection, including typical job tasks and use of personal protective equipment (PPE),^¶^ and to describe illnesses among seropositive persons.

## Methods

### Population Investigated

Field staff members collected anonymized serum specimens and conducted interviews with a convenience sample of farmworkers during June–August 2024. To be eligible, persons had to work on dairies with herds with laboratory-confirmed infection with HPAI A(H5) viruses within the previous 90 days and to have reported no illness on the day of specimen collection.** In Michigan, dairy workers were invited to a central location to participate or offered dairy farm visits; in Colorado, teams visited three dairy farms and invited on-site participation. The interview tools used by MDHHS and CDPHE were adapted from public materials available online.^††^ Interviews^§§^ were conducted in English and Spanish among workers from multiple affected dairies.^¶¶^ This activity was reviewed by CDC, CDPHE, and MDHHS, deemed not research, and was conducted consistent with applicable federal law and CDC policy.***

### Laboratory Methods

Serum specimens were tested at CDC laboratories^†††^ for evidence of recent infection with HPAI A(H5) virus using microneutralization (MN) assays^§§§^ and hemagglutinin inhibition (HI) assays against wild type 2.3.4.4b A/Texas/37/2024 virus.^¶¶¶^ Modified HI assays were conducted using horse erythrocytes optimized for detecting antibodies to A(H5) viruses, as previously described (*1*,*2*). Additional testing was performed on all antibody-positive specimens to eliminate any potential cross-reactivity between antibodies to seasonal influenza viruses and HPAI A(H5) and mitigate concerns about false-positive results (*3*). Serum adsorption was performed on all antibody-positive specimens using a recombinant hemagglutinin head from an influenza A(H1N1)pdm09 virus (A/Wisconsin/588/2019). Geometric mean titers (GMTs) from multiple replicates were calculated to present antibody levels. Persons with a GMT ≥1:40 on both MN and HI assays were considered to have serologic evidence of HPAI A(H5) virus infection; all other results were considered negative. Human specimens were also tested by MN assays against a seasonal influenza A(H1N1)pdm09 virus, A/Victoria/2570/2019.

### Data Analysis

Risk factors for having serologic evidence of HPAI A(H5) infection were assessed; p-values were calculated using Fisher’s exact test. P-values <0.05 were considered statistically significant.

## Results

### Population Characteristics

A total of 115 dairy workers (45 in Michigan and 70 in Colorado) were interviewed and had serum specimens collected; the total number of dairies contacted or workers employed across these dairies was not recorded across states (Table 1). Dairy workers typically spoke Spanish, and 72% of interviews were conducted in Spanish. Specimens were collected at a median of 49 days after first exposure (IQR = 47–59 days) based on the date HPAI A(H5) infection in the herd was confirmed. Among all workers, 21 (18%) reported receipt of the 2023–24 seasonal influenza vaccine.

**TABLE 1:** Characteristics of dairy workers enrolled in serosurveys and potential workplace exposures to highly pathogenic avian influenza A(H5) viruses — Colorado and Michigan, 2024

Characteristic	No. (%)		
	Overall N = 115	Colorado n = 70	Michigan n = 45
Spanish-language survey administered*	**83 (72)**	63 (90)	20 (44)
No. of days since first exposure,^†^ median (IQR)	**49 (47–59)**	48 (47–49)	61 (44–84)
Received 2023–2024 seasonal influenza vaccination^§^	**21 (18)**	13 (19)	8 (18)
**Job tasks after cows became ill**			
No. of job tasks (IQR)	**5 (2–8)**	4 (2–6)	6.0 (4–9)
Breeding cows	**30 (26)**	12 (17)	18 (40)
Changing or cleaning bedding	**38 (33)**	18 (26)	20 (44)
Checking milk quality	**32 (28)**	19 (27)	13 (29)
Cleaning the milking parlor	**49 (43)**	28 (40)	21 (47)
Feeding cows	**46 (40)**	27 (39)	19 (42)
Helping with calving	**43 (37)**	16 (23)	27 (60)
Milking cows	**68 (59)**	36 (51)	32 (71)
Moving or hauling cattle	**56 (49)**	33 (47)	23 (51)
Moving or hauling milk	**15 (13)**	7 (10)	8 (18)
Removing manure or dung	**71 (62)**	37 (53)	34 (76)
Vaccinating cows	**51 (44)**	26 (37)	25 (56)
Working in maternity pens	**49 (43)**	23 (33)	26 (58)
Working with calves	**46 (40)**	22 (31)	24 (53)
**Reported contact with cows with avian influenza^¶^**			
Yes	**69 (60)**	31 (44)	38 (84)
No or unknown	**46 (40)**	39 (56)	7 (11)
**Other animal exposures reported**			
Cats	**31 (27)**	10 (14)	21 (47)
Dogs	**22 (19)**	9 (13)	13 (29)
Pigs	**1 (0.9)**	0 (—)	1 (2.2)
Poultry	**12 (10)**	4 (5.7)	8 (18)
Rodents	**7 (6.1)**	1 (1.4)	6 (13)
Wild birds	**9 (7.8)**	2 (2.9)	7 (16)
Other (sheep, goats, horses, and deer)	**8 (7.0)**	2 (2.9)	6 (13)
Use of PPE**			
Apron	**25 (22)**	14 (20)	11 (24)
Boots or boot covers	**70 (61)**	31 (44)	39 (87)
Coveralls	**29 (25)**	12 (17)	17 (38)
Gloves	**75 (65)**	33 (47)	42 (93)
Head or hair cover	**48 (42)**	29 (41)	19 (42)
N95 or other respirator	**24 (21)**	10 (14)	14 (31)
Safety goggles	**42 (37)**	27 (39)	15 (33)
**Use of non-PPE items**			
Bandana or gaiter	**16 (14)**	13 (19)	3 (7)
Sunglasses	**21 (18)**	11 (16)	10 (23)
Other type of mask	**14 (20)**	14 (20)	0 (—)
**Consumption of raw dairy products**	**11 (10)**	6 (9)	5 (11)

**Abbreviation:** PPE = personal protective equipment.

* Spanish compared with English; interviews were available in other languages using real-time translation services, but only Spanish and English interviews were conducted.

^†^ Defined as the worker-reported date when cows first started showing symptoms of bird flu at this dairy (in Michigan) or the quarantine date (in Colorado).

^§^ One person in Colorado reported unknown influenza vaccination status.

^¶^ Persons were asked, “Did you ever work with cows that were sick with bird flu?” (Michigan). Alternatively, persons were asked if they were within 6 feet of cows that were ill and then, “Were any of these cows known or suspected to have bird flu?” (Colorado). Persons who answered “yes” to either of these questions were reported as having worked with cows with bird flu, and other responses were combined.

** Persons were asked about use of coveralls, safety goggles, gloves, waterproof aprons, sunglasses, bandanas or gaiters, N95 masks or other respirators, head or hair covers, rubber boots or boot covers, or other PPE. In Colorado, persons were also asked about use of other types of masks. Persons were asked if they wore this PPE “after cows started to get sick” (Michigan) or asked if they used this PPE since the week after the quarantine date (Colorado).

Workers reported multiple job tasks; those most frequently reported included cleaning manure (62%), milking cows (59%), and moving or hauling cattle (49%). A minority of workers reported close contact with other animal species in which HPAI A(H5) clade 2.3.4.4.b viruses might have been circulating, including cats (27% of workers), poultry (10%), and wild birds (8%). After infection was detected in cows, a minority of workers reported use of CDC-recommended PPE for eye protection (37% reported use of safety goggles) or respiratory protection (21% reported use of N95**** or other respirators).^††††^

### HPAI A(H5) Virus Seroprevalence

Among the 115 dairy workers, eight (7%; 95% CI = 3.6%–13.1%) had serologic evidence of infection with A(H5) virus (both neutralizing antibody titers and HI antibody titers ≥1:40) (Table 2). Overall, 78 (66%) workers had neutralizing antibody titers ≥1:40 against seasonal influenza A(H1N1)pdm09 virus, suggesting previous vaccination or infection with seasonal influenza A(H1N1)pdm09 virus. All persons with a positive serology result were Spanish speakers, all reported cleaning the milking parlor, and most (88%) reported milking cows. Among those with negative results, 70% were Spanish speakers, 38% reported cleaning the milking parlor, and 57% reported milking cows. Cleaning the milking parlor was the only task significantly associated with a positive test result (p<0.001). None of the workers with serologic evidence of infection used respiratory protection; three used recommended eye protection. Among the eight workers with evidence of infection, only one reported close contact with cows known to be infected,^§§§§^ compared with 68 (64%) workers with negative test results. However, all worked on farms with herds that were reported to public health officials as being HPAI A(H5)–infected.

**TABLE 2:** Potential risk factors for serologic evidence of infection with highly pathogenic avian influenza A(H5) among dairy workers (N = 115) — Colorado and Michigan, 2024

Characteristic	No. (%)		
	Seronegative n = 107; 93% of total	Seropositive n = 8; 7% of total	p-value*
**Spanish-language survey**	75 (70)	8 (100)	0.10
**State**			
Colorado	64 (60)	6 (75)	0.5
Michigan	43 (40)	2 (25)	—
**No. of days since exposure, median (IQR)**	49 (47–59)	49 (49–51)	>0.9
**Antibody titers**			
HI GMT: influenza A, H5^†^ median (IQR)	5 (5–5)	49 (40–80)	—
MN GMT: influenza A, H5^†^ median (IQR)	5 (5–10)	49 (40–63)	—
MN titers: seasonal influenza A, H1^§^ median (IQR)	80 (20–320)	30 (18–110)	—
**Seasonal flu vaccination received** ^¶^	20 (19)	1 (13)	>0.9
**Job tasks after cows became ill**			
Breeding cows	29 (27)	1 (13)	0.7
Changing or cleaning bedding	36 (34)	2 (25)	>0.9
Checking milk quality	28 (26)	4 (50)	0.2
Cleaning the milking parlor	41 (38)	8 (100)	<0.001
Feeding cows	45 (42)	1 (13)	0.14
Helping with calving	40 (37)	3 (38)	>0.9
Milking cows	61 (57)	7 (88)	0.14
Moving or hauling cattle	53 (50)	3 (38)	0.7
Moving or hauling milk	13 (12)	2 (25)	0.3
Number of job tasks, median (IQR)	5 (2–8)	5 (3–7)	0.7
Removing manure or dung	66 (62)	5 (63)	>0.9
Vaccinating cows	47 (44)	4 (50)	>0.9
Working in maternity pens	46 (43)	3 (38)	>0.9
Working with calves	44 (41)	2 (25)	0.5
Reported contact with cows with bird flu**			
Yes	68 (64)	1 (13)	0.007
No or unknown	39 (36)	7 (88)	—
**Other animal exposures reported**			
Cats	30 (28)	1 (13)	0.7
Dogs	22 (21)	0 (—)	0.3
Pigs	1 (1)	0 (—)	>0.9
Poultry	12 (11)	0 (—)	>0.9
Rodents	6 (6)	1 (13)	0.4
Wild birds	8 (8)	1 (13)	0.5
Other (sheep, goats, horses, or deer)	7 (7)	1 (13)	0.4
**Use of PPE^††^**			
Apron	23 (21)	2 (25)	>0.9
Boots or boot covers	66 (62)	4 (50)	0.7
Coveralls	29 (27)	0 (—)	0.2
Gloves	70 (65)	5 (63)	>0.9
Head or hair cover	45 (42)	3 (38)	>0.9
N95 or other respirator	24 (22)	0 (0)	0.2
Safety goggles	39 (36)	3 (38)	>0.9
**Consumption of raw dairy products**	11 (10)	0 (—)	>0.9

**Abbreviations:** GMT = geometric mean titer; HI = hemagglutinin inhibition assay; MN = microneutralization assay; PPE = personal protective equipment.

* P-values were calculated using Fisher’s exact test.

^†^ Influenza A, H5 virus antibody titers were generated using influenza A/Texas/37/2024 virus, a wild-type virus isolated from the March 2024 human infection in Texas.

^§^ Seasonal influenza A, H1 virus titers were generated using A/Victoria/2570/2019, a virus similar to both circulating influenza A, H1N1 viruses, and the vaccine strain.

^¶^ Seasonal influenza vaccination was unknown for one seronegative person.

** Persons were asked, “Did you ever work with cows that were sick with bird flu?” (Michigan). Alternatively, persons were asked if they were within 6 feet of cows that were ill and then, “Were any of these cows known or suspected to have bird flu?” (Colorado). Persons who answered “yes” to either of these questions were reported as having worked with cows with bird flu, and other responses were combined.

^††^ Persons were asked about use of coveralls, safety goggles, gloves, waterproof aprons, sunglasses, bandanas or gaiters, N95 masks or other respirators, head or hair covers, rubber boots or boot covers, or other PPE. In Colorado, persons were also asked about use of other types of masks. Persons were asked if they wore this PPE “after cows started to get sick” (Michigan) or asked if they used this PPE since the week after the quarantine date (Colorado).

### Illness and Seropositivity to HPAI A(H5) Virus

Among all 115 dairy workers, 46 (40%) reported feeling ill shortly before or during the period that A(H5) virus infection was confirmed in cows on the farms where they worked (Table 3). Four of these illnesses were among the eight workers with serologic evidence of infection; among these persons, signs and symptoms most frequently reported were red, draining, or itching eyes (three). These signs and symptoms were also frequently reported among workers who were ill but who had negative HPAI A(H5) serology (26 of 42; 62%). Among the four workers with positive test results, feverishness, sore throat, runny or stuffy nose, sneezing, diarrhea, and headache were each reported by one worker; these signs and symptoms were also reported by persons with negative serology results. Among persons with serologic evidence of infection, illness onset occurred a median of 5 days before the date of detection of HPAI A(H5) virus among cows within the dairy where they worked.

**TABLE 3:** Characteristics of illnesses reported by dairy workers, by seropositivity to highly pathogenic avian influenza A(H5) (N = 115) — Colorado and Michigan, 2024

Reported signs and symptoms*	Serologic test result, no. (%)	
	Negative n = 107	Positive n = 8
**Any self-reported illness**	42 (39)	4 (50)
**No. of days from exposure**^†^ **to onset, median (IQR)**	15 (4 to 27)	–5 (–11 to 1)
Cough	13 (31)	0 (—)
Diarrhea	6 (15)	1 (25)
Difficulty breathing	7 (17)	0 (—)
Fatigue	21 (50)	0 (—)
Fever (≥100.4°F [≥38°C])	7 (17)	0 (—)
Feverishness or chills	15 (37)	1 (25)
Headache	19 (45)	1 (25)
Muscle aches	19 (45)	0 (—)
Nausea or vomiting	4 (9.5)	0 (—)
Rash	4 (9.5)	0 (—)
Red, draining, or itching eyes	26 (62)	3 (75)
Runny nose or nasal congestion	20 (48)	1 (25)
Seizure	0 (—)	0 (—)
Sneezing	13 (31)	1 (25)
Sore throat	24 (57)	1 (25)

* Defined as an affirmative response to the question, “Since cows have started to get sick, have you been sick” (Michigan) or “Since [the date of detection per farm], did you develop any symptoms?” (Colorado). Individual symptoms were then elicited, including fever (measured ≥100.4°F [≥38°C]), feverishness/chills, cough, fatigue or tiredness/sluggishness, sore throat, runny or stuffy nose, sneezing, nausea/vomiting, diarrhea, headache, rash, muscle/body aches, red/draining or itching eyes, difficulty breathing/shortness of breath, or seizures. Symptoms were only elicited among persons who reported illnesses.

^†^ Defined as the worker-reported date when cows first began showing symptoms of bird flu on this dairy farm (Michigan), or the quarantine date (Colorado).

## Discussion

In this analysis, 7% of exposed dairy farm workers in Michigan and Colorado had serologic evidence of infection with HPAI A(H5). These data reaffirm the importance of identifying and implementing interventions to prevent dairy cattle infections to reduce worker exposure and using infection prevention measures among farm workers when HPAI A(H5) virus infection is confirmed or suspected in a herd.^¶¶¶¶^ Before the emergence of clade 2.3.4.4.b viruses, estimates of anti-HPAI A(H5) seroprevalence among workers exposed to infected poultry were approximately 0%–0.6% globally (*4*) and approximately 4.6% in Egypt after the emergence of clade 2.3.4.4.b viruses in poultry (*5*). Preliminary data available from a single dairy in the United States showed that two of 14 exposed workers had elevated neutralizing antibodies against HPAI A(H5) (*6*). These data from Michigan and Colorado provide the largest sample to date, estimating the risk to dairy farm workers associated with the ongoing cattle epizootic.

Among workers who had antibodies to HPAI A(H5) virus, all (100%) reported cleaning the milking parlor, compared with 38% of workers without HPAI A(H5) virus antibodies. Cleaning the milking parlor might be a higher-risk workplace activity given the high HPAI A(H5) viral load in the milk of infected cows (*7*). None of the workers with HPAI A(H5) virus antibodies reported using the PPE recommended for working with HPAI A(H5)–infected animals, and use of recommended PPE was low among all workers (*8*). These findings support the need for improved outreach to employers and workers about the risk for infection when working with dairy cattle infected with HPAI A(H5) viruses, and for the use of infection prevention measures such as PPE (*8*). Only one of the persons whose test results indicated antibodies to HPAI A(H5) virus reported working with known HPAI A(H5) virus–infected cows, supporting the need for additional education and outreach to employers and farm workers once HPAI A(H5) is identified in herds. Because most workers (and all those with positive serology results) spoke Spanish, this outreach should be culturally appropriate (*9*) and delivered in the workers’ spoken languages. Approximately 80% of the dairy workers from this investigation population might also benefit from outreach offering seasonal influenza vaccination.

One half of the persons with antibodies to HPAI A(H5) virus did not report illness; asymptomatic infection has been observed in past HPAI A(H5) serologic investigations (*4*). Some of the persons who did not report being ill might have experienced only very mild symptoms. This finding highlights the need to actively monitor exposed workers by assessing the presence of any mild symptoms and provide a safe environment that encourages reporting of even mild illness and allows for rapid treatment with antivirals to prevent progression to severe disease, without risk for repercussions in terms of job security and pay *(**8**)*. Some of the persons with antibodies to HPAI A(H5) virus reported illnesses before herds were identified, underscoring the need for early outreach to dairy workers and rapid identification of herds as through expanded herd testing***** and bulk milk testing programs.^†††††^

### Limitations

The findings in this report are subject to at least five limitations. First, enrolled persons volunteered to participate; therefore, this sample might not be representative of all farmworkers. Second, no demographic or medical history data were collected to examine host factors associated with infection. Third, the fraction of HPAI A(H5) infections that are completely asymptomatic might be lower than the frequency of persons with positive serologic results who did not report illness in this report, because of perceptions of mild or subclinical illness and inability to recall. Fourth, PPE questions were not cross-referenced with specific job duties, limiting inferences that can made about PPE effectiveness. Finally, some persons with negative serologic results might have been infected but failed to mount detectable antibody responses for a variety of reasons.

### Implications for Public Health Practice

Primary prevention of HPAI A(H5) virus infections in animals, including dairy cows, is critical to reducing the risk for human infection and mitigating changes in the virus that could lead to a potential HPAI A(H5) pandemic. During the period cattle are infected, employers can reduce the risk for worker infection by following CDC recommendations for engineering controls, worker education on the proper use of PPE, other administrative controls (e.g., testing animals for HPAI A(H5) and developing plans to monitor workers for illness), and providing appropriate PPE to workers (*8*). This investigation identified low PPE adherence among dairy workers, which has been an ongoing challenge in hot, tight spaces where visibility around large animals is important and the use of eye protection can be challenging (*10*). Increased use of PPE might be achieved through adapting current recommendations to meet the needs of dairy farm workers such as simplifying messaging and focusing on highest risk activities (*10*). Employers should prioritize implementation of controls in hot work environments (e.g., worker training acclimatizing protocols, and work/rest schedules) to minimize heat exposures and heat injuries while wearing PPE.^§§§§§^ Another challenge in these environments with significant sources of particulate matter and bioaerosols (e.g., dirt, feces, and milk), is that mild irritation of eyes or the respiratory tract can occur frequently; a low threshold for reporting mild symptoms and seeking testing should be encouraged to identify whether these mild symptoms are caused by HPAI A(H5) virus. Public health practitioners should modify messaging to address the unique setting of exposed dairy workers to identify and treat all HPAI A(H5) virus infections , including mild infections. Finally, data from additional serosurveys could identify additional risk factors for infection and continue refinement of best practices for prevention.

## Footnotes

*
https://www.cdc.gov/bird-flu/php/technical-report/h5n1-06052024.html

†
https://emergency.cdc.gov/han/2024/han00506.asp

§ https://www.michigan.gov/mdhhs/inside-mdhhs/newsroom/2024/05/30/h5n1-updates; https://cdphe.colorado.gov/press-release/colorado-state-health-officials-identify-a-human-case-of-avian-flu; https://www.cdph.ca.gov/Programs/OPA/Pages/NR24-028.aspx

¶
https://www.cdc.gov/bird-flu/spotlights/hpai-health-recommendations.html

** Interviews and blood collections were targeted to occur within 14–90 days of the first highly pathogenic avian influenza A(H5) positive result for each dairy.

††
https://www.cdc.gov/bird-flu/media/pdfs/2024/07/CDC-H5-Epidemiologic-Investigation-Protocol-Materials.pdf

§§ Interviews included reports of symptoms or feeling ill around the time of first exposure to ill cows at the farm on which the person worked; job tasks; self-reported contact with cows that were ill with bird flu; use of PPE; exposure to other animals; and consumption of raw milk or raw milk products.

¶¶ To preserve anonymity, the number of participating dairies and the number of total workers employed at those dairies were not tracked in Michigan.

*** 45 C.F.R. part 46.102(l)(2), 21 C.F.R. part 56; 42 U.S.C. Sect. 241(d); 5 U.S.C. Sect. 552a; 44 U.S.C. Sect. 3501 et seq.

††† Testing was conducted in biosafety level III–enhanced laboratories.

§§§
https://iris.who.int/bitstream/handle/10665/44518/9789241548090_eng.pdf

¶¶¶ Influenza A/Texas/37/2024 was isolated from the human case of highly pathogenic avian influenza A(H5) identified in Texas in 2024 and is antigenically similar to viruses that circulated among dairy cows in Michigan and Colorado in the summer of 2024.

**** N95 is a certification mark of the U.S. Department of Health and Human Services registered in the United States and several international jurisdictions.

†††† Defined as the worker-reported date when cows first started showing symptoms of bird flu on this dairy (Michigan) or the quarantine date (Colorado).

§§§§ Persons were asked, “Did you ever work with cows that were sick with bird flu?” (Michigan). Alternatively, persons were asked if they were within 6 feet of cows that were ill and then, “Were any of these cows known or suspected to have bird flu?” (Colorado). Persons who answered “yes” to either of these questions were reported as having worked with cows with bird flu, and other responses were combined.

¶¶¶¶
https://www.aphis.usda.gov/sites/default/files/recommendations-hpai-livestock.pdf

*****
https://www.aphis.usda.gov/sites/default/files/vs-hpai-dairy-herd-status-program.pdf

†††††
https://www.aphis.usda.gov/news/agency-announcements/usda-builds-actions-protect-livestock-public-health-h5n1-avian-influenza

§§§§§
https://www.cdc.gov/bird-flu/prevention/worker-protection-ppe.html
